# Hyaluronic Acid vs. PRP, PRF, and Collagen in Regenerative Endodontics: A Systematic Review

**DOI:** 10.3390/jcm15114257

**Published:** 2026-05-31

**Authors:** Aneeqah Maryam Farah Ahmad, Tawfiq Hijazi Alsadi, Agustina Muñoz Rodríguez, Kais Hijazi Muwaquet, Susana Muwaquet Rodriguez

**Affiliations:** 1 Department of Dentistry, Faculty of Medicine and Health Science, Catholic University of Valencia, 46001 Valenci, Spain; 2 Department of Orthodontics, Faculty of Medicine and Health Science, Catholic University of Valencia, 46001 Valencia, Spain; 3 Department of Preventive Dentistry and Epidemiology, Faculty of Medicine and Health Science, Catholic University of Valencia, 46001 Valencia, Spain; 4 Department of Restorative Dentistry and Endodontics, Faculty of Medicine and Health Science, Catholic University of Valencia, C/Quevedo, 2, 46001 Valencia, Spain

**Keywords:** collagen, hyaluronic acid, immature necrotic permanent teeth, platelet-rich fibrin, platelet-rich plasma, regenerative endodontics

## Abstract

**Background**: Regenerative endodontic procedures (REPs) aim to restore pulp vitality and promote root development in immature necrotic permanent teeth. Scaffold materials provide a 3D framework to support cellular migration, proliferation, and differentiation and play a critical role in regenerative interventions. Commonly used scaffolds include PRP, PRF, and collagen; however, hyaluronic acid has also demonstrated promising treatment outcomes. **Objective**: To evaluate whether hyaluronic acid (HA) provides superior regenerative outcomes compared to PRP, PRF, and collagen scaffolds. **Methods**: A systematic electronic search was conducted across PubMed, Scopus, and EBSCOhost. A total of 952 articles were identified in the initial search, of which 19 articles were included in the final review. Due to heterogeneity, a narrative synthesis was performed. **Results**: PRF demonstrated the most consistent improvement in root development and dentinal wall thickening. Apical closure and periapical healing were achieved across all scaffold types. Evidence for HA was limited, with no clear superiority identified. **Conclusions**: Current evidence does not support the superiority of HA over established scaffolds. Further standardised clinical trials are required to make definitive comparisons on scaffold effectiveness.

## 1. Introduction

Pulp necrosis refers to the biological death of the tooth and arises from several factors, primarily microbial infection, causing major complications in immature permanent teeth, which can ultimately result in tooth loss [1,2]. Immature teeth are particularly susceptible to fracture due to thin dentinal walls, consequently lowering the survival rate [3]. However, there is a strong capability of immature teeth to heal, highlighting the importance of appropriate management in these cases to ensure maximum chance of tooth development and survival [4]. Regenerative endodontic procedures (REPs) are a proposed solution, gaining popularity, to help restore immature necrotic permanent teeth by inducing apical healing, allowing continued development, and restoring normal physiological function [4,5]. Traditionally, immature necrotic teeth have been managed through apexification techniques, where the primary goal is induction of a calcified apical barrier, allowing the tooth to be sealed effectively [6]. Despite this, a major limitation of this treatment approach is that it does not permit continued root development, which can lead to failure due to weak tissue structure. For these reasons, alternative biologically-based treatments are required to enhance the long-term survival of immature necrotic permanent teeth.

Regenerative endodontics is defined as “biologically based procedures designed to replace damaged tooth structures, including the dentine and root structures, as well as cells of the pulp-dentine complex” [7]. The objective of the procedure is to regenerate pulp tissue by exploiting natural healing mechanisms to facilitate continued root maturation, addressing the limitations of apexification [8]. REPs rely on three main factors for success: scaffolds, stem cells, and growth factors, which combined play an essential role in tissue engineering [8,9].

Stem cells of the dental pulp and apical papilla are required for the repair and formation of dental tissues, while growth factors are active signalling molecules that help regulate their proliferation and differentiation. Scaffolds provide a three-dimensional (3D) framework needed for stem cell migration and adhesion within the canal system. This provides a structure to support growth and can obtained from various sources [8,9].

There are three main objectives of regenerative endodontic procedures defined by the American Association of Endodontists. The primary objective is the elimination of symptoms and evidence of bony healing. The secondary goal is increased root wall thickness and/or length. Finally, restoration of a positive vitality response is the tertiary goal, despite this outcome being less consistently reported [10,11,12].

As previously mentioned, scaffolds are a fundamental component of pulpal regeneration. They fulfil two main roles: providing 3D structural support to enhance cellular organisation and stabilise the blood clots and serving as a biomimetic model of the pulp extracellular matrix. Through the controlled release of growth factors and bio-signalling, scaffolds promote cell proliferation and differentiation, enhancing odontogenic tissue formation. Moreover, they play a crucial role in facilitating cell homing by re-creating the appropriate regenerative microenvironment [12,13]. An ideal scaffold should be biocompatible and biodegradable, and it should provide adequate mechanical and physical strength and promote cell adhesion within a stable 3D structure [12,13]. Various intracanal scaffolds have been proposed and generally classified by origin into natural, synthetic, or host-derived [8,13].

Traditionally, blood clots (BCs) are the most common scaffolds used in REPs, forming a fibrin network that supports stem cell migration and acts as a reservoir of growth factors, promoting cell proliferation and differentiation. Despite their ease of clinical application, economic factors, and biocompatibility, limitations include insufficient canal filling, mechanical stability, and unpredictable outcomes [13,14].

Platelet-rich plasma (PRP) is classified as an autologous platelet concentrate (APC) and is considered the first-generation form of this scaffold group [14]. PRP serves as a reservoir of bioactive molecules, promoting tissue healing through cell interaction, proliferation, and differentiation [13,15]. However, its two-stage preparation method is technique-sensitive, which has led to inconsistent results in experimentation [13,16].

On the other hand, platelet-rich fibrin (PRF) is a second-generation APC, prepared in a single stage reducing the biochemical manipulation of blood [15]. PRF forms a 3D biological framework that facilitates cell migration and proliferation through a sustained release of growth factors at a slower rate, rendering it an efficient scaffold alternative [13]. Whilst these scaffolds are simpler to prepare, the lack of standardisation in preparation protocols remains a challenge [8].

Collagen is a naturally occurring biomaterial derived from the extracellular matrix that supports cell adhesion and organisation within the canal system. The biodegradable and biocompatible scaffold can be provided in various forms, with increased viscoelastic properties; however it also presents a lack of mechanical strength and unpredictable degradation rates [13,17,18].

Hyaluronic acid (HA) is a glycosaminoglycan that has gained interest as a scaffold in regenerative endodontics due to its biocompatibility, biodegradability, and promotion of angiogenesis and odontogenesis. As a natural constituent of the extracellular matrix, it can be used in several forms including hydrogels and sponges. Although it has been reported to enhance cellular recruitment and influence stem cell expansion, it is associated with limited mechanical stability and hypersensitive reactions [13,17,19]. The hydrogel form allows the scaffold to adapt to the morphology of the root canal, providing flexibility and elasticity, with studies demonstrating preserved cell viability and proliferation, although current evidence is mainly limited to in vitro and pre-clinical studies [20,21].

Numerous scaffolds have been proposed in regenerative endodontics; however, they present differences in mechanical properties, biological activity, and clinical applications, which may influence regenerative outcomes including root development. Despite increasing interest in these materials, direct comparative clinical evidence remains limited.

These differences are of particular importance when evaluating scaffold effectiveness in regenerative endodontic treatment, as explored in this review.

## 2. Aim

This review aims to evaluate whether hyaluronic acid provides superior clinical and radiographic regenerative outcomes compared to other scaffold materials, such as platelet-rich plasma, platelet-rich fibrin, and collagen, in immature necrotic permanent teeth.

## 3. Objectives

This study aims to compare the effectiveness of hyaluronic acid with PRP, PRF, and collagen scaffolds in regenerative endodontic procedures in necrotic immature permanent teeth based on clinical and radiographic outcomes. A narrative synthesis was conducted to analyse and interpret findings to provide a conclusion on the regenerative endodontic outcomes of scaffold materials.

More specifically this review seeks to compare the effect of different scaffolds (HA, PRP, PRF, collagen) on root development, including length and dentinal wall thickening, and to evaluate differences in apical closure and periapical healing among scaffold types. It also aims to assess clinical outcomes, including symptom resolution and return of pulp vitality and to analyse the consistency and statistical significance of reported outcomes across studies.

## 4. Materials and Methods

### 4.1. Study Design

The present study protocol was registered in Open Science Framework (https://osf.io/snr9d, accessed on 27 April 2026) and to ensure methodological transparency and reproducibility, this systematic review was developed according to the Preferred Reporting Items for Systematic Reviews and Meta-Analyses (PRISMA) 2020 guidelines. The completed PRISMA 2020 checklist is provided in Supplementary Materials (Table S1). The review assessed how the use of different scaffold materials, including hyaluronic acid, PRP, PRF, and collagen, relates to regenerative endodontic outcomes such as root development, apical closure, and periapical healing through the analysis of clinical and radiographic data from the included studies.

The following PICO question comprising four elements was constructed for the review.

P (population): patients with immature necrotic permanent teeth.
I (intervention): endodontic regeneration with hyaluronic acid scaffold.
C (comparison): endodontic regeneration with PRP, PRF, and collagen scaffolds.
O (outcome): endodontic treatment outcomes: Root development, Apical closure, Periapical healing.

The PICO question was as follows: “In patients with immature necrotic permanent teeth, does the use of hyaluronic acid as a scaffold compared to PRP, PRF or collagen scaffolds result in improved regenerative endodontic outcomes such as root development, apical closure and periapical healing?”

### 4.2. Eligibility Criteria

The studies included adhered to the predefined conditions stated in the eligibility criteria below.

The inclusion criteria:

Study Type: Human studies published within the last 10 years (between 2015 and 2025) reporting regenerative endodontic procedures, studies reporting quantitative or qualitative outcomes of success, studies with minimum follow-up of ≥6 months after treatment. Comparative clinical studies (RCTs, controlled trials, cohort studies) were included only if they contained a comparison group investigating two or more scaffolds.

Population: Immature permanent teeth with necrotic pulp.

Type of Intervention: Regenerative endodontic procedures using one or more of the following intracanal scaffolds: HA, PRP, PRF, and collagen. Blood clot was included as a conventional control scaffold.

Type of Outcome Variables: Studies reporting at least one regenerative outcome including root length, dentin wall thickening, apical closure, periapical healing, return of sensibility or vitality, or resolution of clinical signs (absence of pain, swelling, or reinfection).

Exclusion criteria:

Study Type: Animal studies, in vitro studies, systematic reviews, meta-analyses, case reports and case series, studies not reporting clinical or radiographic outcomes, non-peer reviewed papers, abstracts, and letters or conference.

Population: Primary teeth, mature teeth (closed apex).

Type of Intervention: Purely biomechanical scaffolds; regenerative procedures without scaffolds, or blood-clot-only protocols without a comparison group.

Type of Outcome Variables: Data from studies that have been conducted in vitro; studies evaluating stem cell viability; studies only reporting histological or cellular outcomes.

Studies were not excluded based on journal indexing status to ensure comprehensive inclusion of relevant evidence. Methodological quality and risk of bias were assessed using validated tools, therefore inclusion of studies with unclear indexing was justified based on their relevance and acceptable quality.

### 4.3. Search Strategy and Study Selection

An electronic search was conducted in December 2025 across three databases: PubMed, EBSCOhost (all available databases), and Scopus. Combining the appropriate MeSH terms and Boolean operators with the keywords derived from the constructed PICO question, multiple search strategies were performed in each database, to ensure comprehensive retrieval of relevant studies in Table 1.

A complementary manual search was also performed in Google Scholar to screen grey literature. Three search strategies were conducted, and the first 200 results from each were screened in accordance with relevance-based ranking, resulting in 600 records.

The search methodology was developed to identify clinical trials investigating HA, PRP, PRF, and collagen. Among the records identified, those comparing the experimental scaffolds to blood clots as a conventional control were included to account for the limited available data.

The search outcomes were transferred to a data management tool to remove duplicate entries. The initial stage of screening was conducted using the article title and publication year to include or exclude trials, followed by a second stage assessing the abstract. The final studies were evaluated comprehensively by reading the full text available. The screening and eligibility assessment were conducted by two reviewers (A.M.F.A. and S.M.R.), according to the predefined inclusion and exclusion criteria, to minimise selection bias. Any disagreements were resolved through discussion until a consensus was reached. The inter-reviewer agreement during the screening process was assessed using Cohen’s Kappa coefficient, obtaining a κ-value = 0.85, which falls between the range of 0.80 < κ < 1.00 indicating ‘very good’ agreement.

### 4.4. Data Extraction

The data extraction was performed by two reviewers, using a standardised approach to compile the relevant findings from all included studies. The following variables from each study were reported in a master table:

- Study characteristics: author, year of publication, country, study design.
- Sample characteristics: age, sample size, number of teeth treated, and tooth type.
- Intervention characteristics: scaffold material used and disinfection protocol (irrigant).
- Follow-up characteristics: follow-up duration.
- Outcome measures: root length increase, dentinal wall thickening, apical closure, periapical healing, return of vitality, and clinical success.
- Radiographic assessment details: imaging method, radiographic standardisation protocol, and measurement software/tool used.
- Methodological characteristics: blinding procedures, statistical tests used, and reported significance levels.

The extracted data was then used for qualitative synthesis and quantitative comparison where appropriate.

### 4.5. Data Synthesis

Meta-analysis was first considered for this review; however, following the data extraction, it was ruled unfeasible due to significant heterogeneity among studies and the scarce number of HA trials available. Variability was observed in the measurement techniques, degree of radiographic standardisation, reporting of examiner blinding, and reliability and outcome assessment methods. The lack of sufficient comparable statistical data, coupled with the methodological and clinical heterogeneity, limited the direct comparison across the included studies. Therefore, a narrative synthesis approach was performed where outcomes were grouped into clinical and radiographic features and results were compared by scaffold type to identify patterns in regenerative success.

The outcomes assessed in this review were categorised into primary and secondary outcomes. The primary outcome assessed was root length increase, while secondary outcomes included dentinal wall thickening, apical closure, periapical healing, and clinical success. These outcomes were evaluated using clinical examination findings and radiographic assessment methods including periapical radiographs and, where available, cone-beam computed tomography (CBCT).

### 4.6. Quality and Risk of Bias Assessment

A risk of bias assessment was carried out on all included studies using the Cochrane ROBINS-I tool for non-randomised studies and the Cochrane RoB2 tool for randomised study designs.

Additionally, a GRADE assessment was performed to evaluate the certainty of evidence for each outcome reported in the studies included.

Furthermore, the PRISMA 2020 statement was followed to ensure transparent reporting of this systematic review.

## 5. Results

### 5.1. Selection of Studies Using PRISMA Flow Chart

From the initial search a total of 952 articles were collected from the following databases: PubMed (*n* = 209), EBSCOhost (*n* = 114), Scopus (*n* = 29), and Google Scholar (*n* = 600) and were exported to Microsoft Excel for management.

Before commencing the screening process, all retrieved articles were combined into a single dataset, and duplicate records were identified and removed. Following deduplication, a total of 531 records remained. The initial stage of screening was conducted using the article title and publication year to include or exclude articles that fell into the relevant criteria. A total of 502 articles were not eligible for the research and were removed. The remaining 29 articles underwent the second stage of screening, which consisted of an assessment of the abstract, comprising an overview of the introduction, materials and methods, results, and conclusion. Following this, a total of 10 articles were excluded due to failing to answer the PICO question or not fulfilling the inclusion criteria as well as those that could not be retrieved.

**Figure 1:**
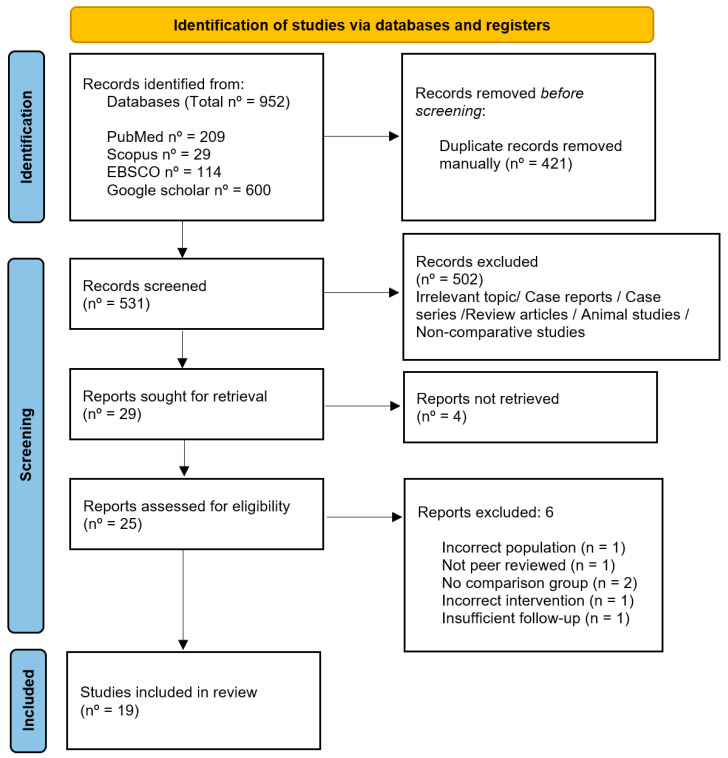
PRISMA 2020 flowchart demonstrating the scheme that was followed in the selection of articles for the review.

The remaining articles were evaluated comprehensively by reading the full text available, resulting in 19 studies included in the final narrative synthesis [22,23,24,25,26,27,28,29,30,31,32,33,34,35,36,37,38,39,40]. The process is outlined below in Figure 1.

### 5.2. Characteristics of Included Studies

A total of 19 studies were included in this review and consisted of different study designs, comprising 15 randomised trials and four non-randomised studies. The selected reports investigated various scaffold materials comprising hyaluronic acid, platelet-rich plasma, platelet-rich fibrin, and collagen, with several studies assessing more than one of the included scaffold types.

A detailed summary of the study characteristics is presented in Table 2.

### 5.3. Measurement Characteristics of Included Studies

Measurement characteristics between the included studies varied significantly. Radiographic assessment outcomes were primarily evaluated using periapical radiographs, whilst some studies also used CBCT during the follow-up periods. Radiographic standardisation techniques were not universally reported. When described, standardisation was followed using positioning devices, paralleling techniques, or radiographic stents to ensure consistent positioning during follow-ups. Outcomes of root length, dentin wall thickening, and apical closure were reported in several forms including quantitatively, qualitatively, or as a categorical score. Additionally, radiographic standardisation procedures differed across reports as well as variations in follow-up duration. Clinical outcomes were inconsistently reported and were commonly presented as binary variables.

Overall, considerable heterogeneity existed between the studies in measurement techniques and outcome reporting, which limited the direct comparison of results; thus, a narrative evaluation was justified.

### 5.4. Clinical and Radiographic Outcome Assessment

#### 5.4.1. Root Length Increase

Eighteen of the included studies assessed root lengthening, and overall, the majority demonstrated an increase following regenerative endodontic procedures, irrespective of scaffold material.

Statistically significant differences between scaffold groups were reported in several studies. PRF frequently demonstrated superior outcomes compared to PRP and blood clots, while PRP also showed significant improvements over blood clots in multiple trials. However, many studies did not report statistically significant differences in root length increase between scaffold groups, indicating variability in outcomes.

Regarding hyaluronic acid, limited evidence was available. One study reported significant differences, with PRF showing the greatest increase, followed by HA and collagen, whilst another study found no significant difference between HA and blood clots.

Overall, PRF appears to provide the most consistent improvement in root length, although evidence for HA remains limited and inconclusive.

#### 5.4.2. Dentinal Wall Thickening

Dentinal wall thickening was reported in 18 of the included studies. In general, an increase in dentinal wall thickness was present with regenerative treatment regardless of the scaffold choice.

Multiple reports identified statistically significant differences between scaffold types. PRF commonly proved more effective than PRP and BCs, while in several studies PRP yielded more favourable results than BCs. In contrast, seven trials reported no statistically significant differences in dentin thickness between scaffold groups, although an increase was observed.

Evidence relating to hyaluronic acid was limited. One study demonstrated significantly greater dentin thickening with HA compared to BCs, whereas another revealed the greatest increase in dentin thickness with PRF followed by HA and collagen respectively.

Overall, PRF demonstrated the most consistent improvement, while HA showed promising but limited evidence.

#### 5.4.3. Apical Closure

All included studies assessed outcomes related to apical closure following regenerative procedures, and collectively, most reported evidence of closure irrespective of scaffold type.

Statistically significant outcomes were reported in various studies. PRF consistently demonstrated higher efficacy in apical closure than PRP and BCs, whilst both collagen and PRP were found to have greater closure when compared to BC in other investigations. The two hyaluronic acid reports also detected significant differences. One study identified greater results with HA compared to BC whilst the other observed a hierarchy of outcomes with superior closure in PRF followed by HA and collagen. Conversely, a substantial number of studies did not report statistically significant differences in apical closure, suggesting inconsistency in evidence.

Overall, all scaffolds promoted apical closure with PRF and HA showing favourable trends.

#### 5.4.4. Periapical Healing

All included investigations reported outcomes of periapical healing, with most observing positive responses regardless of scaffold type.

Only five studies reported statistically significant results in healing following regenerative treatment. Among these, one trial demonstrated superior outcomes with PRP compared to PRF, whilst others showed greater effects with PRF compared to PRP and BCs. Additionally, in certain cases PRP was observed to have greater healing than BC. A few studies documented no statistically significant differences between scaffold groups, while others including those evaluating HA, presented only qualitative findings with general resolution of pathology.

Periapical healing was consistently achieved regardless of scaffold type, suggesting this outcome is less dependent on scaffold selection.

#### 5.4.5. Return of Vitality

The return of vitality following regenerative treatment was reported in only nine of the included studies, with the overall findings varying across the reports.

Studies investigating the outcome identified positive vitality responses in several cases, with multiple identifying a higher proportion of responses with PRP compared to PRF and BCs. Despite these findings, no response or negative vitality at the end of the follow-up period was found in several reports. With respect to hyaluronic acid investigations, vitality responses were not assessed.

In summary return of vitality was inconsistent and cannot be reliably associated with any specific scaffold.

#### 5.4.6. Clinical Success

Clinical success was documented in all studies, with the majority expressing the resolution of symptoms following the regenerative procedure across all scaffold groups. Whilst definitions of success varied across the investigations, most defined it as the resolution of symptoms including pain, fistula, and abscess. Several studies did not specifically define the variable, but it was inferred as the absence of symptoms and radiographic improvements. In relation to hyaluronic acid, a limited number of studies noted that 100% of patients were asymptomatic.

### 5.5. Outcome Summary

Overall, regenerative endodontic procedures resulted in favourable clinical and radiographic outcomes regardless of scaffold type. PRF presented consistently greater outcomes across most variables, particularly in root length increase and dentinal wall thickening, when compared to other scaffolds. PRP demonstrated moderate efficacy and generally outperformed blood clots, while a few studies observed positive outcomes with collagen but with restricted levels of evidence. Hyaluronic acid has exhibited promising results in several outcomes including dentinal wall thickening and apical closure; however, clinical evidence remains extremely limited preventing definitive conclusions from being drawn about its therapeutic value. This table qualitatively summarises the trends identified for the scaffolds investigated in Table 3.

### 5.6. Risk of Bias Assessment

**Figure 2:**
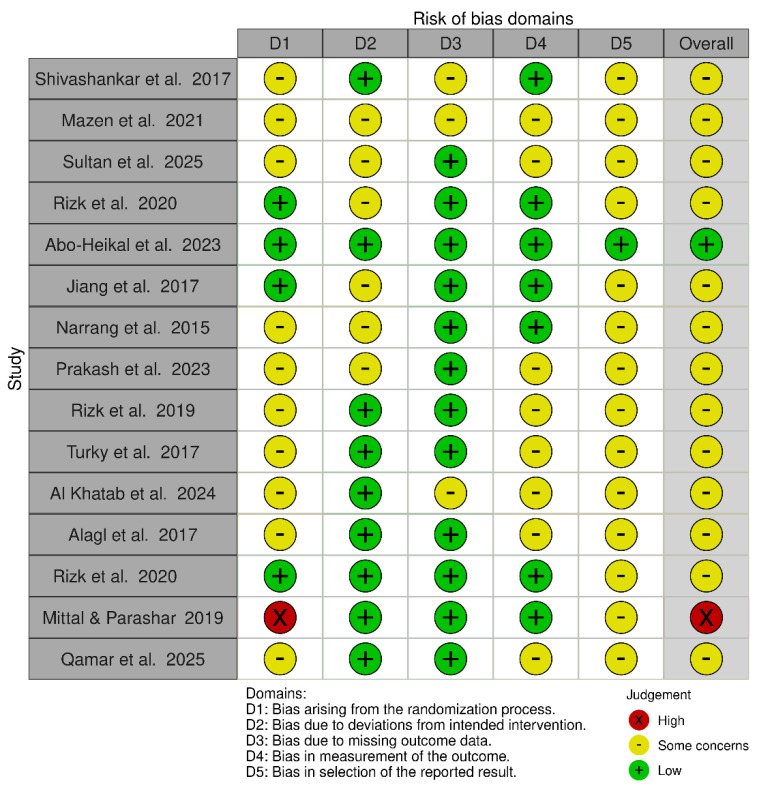
Cochrane RoB2 risk of bias assessment [22,23,24,25,26,27,28,29,32,33,35,36,37,39,40].

**Figure 3:**
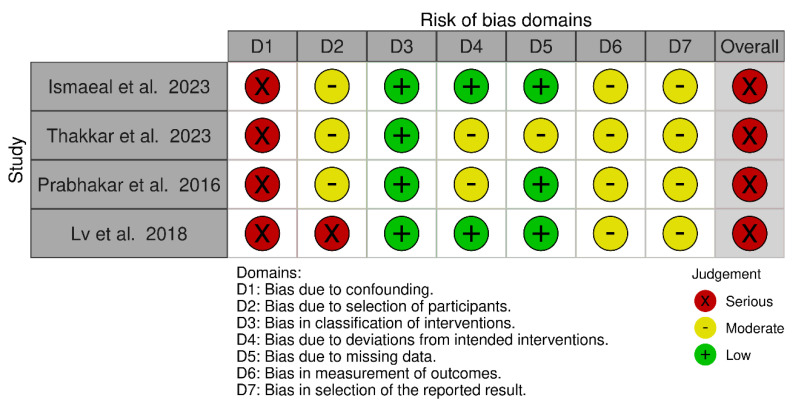
Cochrane ROBINS-I risk of bias assessment [30,31,34,38].

A risk of bias assessment was carried out on all included studies using the Cochrane ROBINS-I tool for non-randomised studies and the Cochrane RoB2 tool for randomised study designs. Among the randomised trials (*n* = 15) (Figure 2), 86.7% were classified as having “some concerns”, with one study reported as high-risk (6.7%) and one as low-risk (6.7%). Therefore, the overall risk of bias for randomised studies was rated as moderate to high-risk. The non-randomised studies (*n* = 4) (Figure 3) were all classified as having “serious risk” of bias (100%), resulting in an overall judgement of serious risk.

GRADE (Table S2) assessment demonstrated that the overall certainty of evidence ranged from low to very low across the outcomes investigated in this review. This is primarily due to heterogeneity in outcome assessment methods, differences in reporting procedures, and methodological limitations.

## 6. Discussion

This systematic review aimed to evaluate if the use of hyaluronic acid as a scaffold in regenerative endodontic procedures results in improved clinical and radiographic outcomes when compared to other scaffolds—PRP, PRF, and collagen.

Generally, most studies suggested that regenerative endodontic treatment using a variety of scaffold materials is associated with successful outcomes, in particular demonstrating evidence of improved root development and periapical healing. Nevertheless, the current available literature does not allow for reliable conclusions to be made on whether superiority is evident between the scaffolds investigated. Although previous literature evaluating scaffold materials involved in REPs exists, direct clinical comparison of HA vs. other scaffolds in immature necrotic permanent teeth remains extremely limited. Due to the insufficient HA evidence, the inclusion and exclusion criteria were broadened to allow for wider comparison across the investigated scaffold types. Therefore, trials assessing alternative scaffold combinations, including PRP, PRF, collagen, and blood clots, as a control scaffold, were also evaluated to provide a more complete review of scaffold performance. However, considerable heterogeneity between the study characteristics and measurement techniques of the included reports limited direct comparisons of results.

PRP and PRF were investigated in a larger number of studies compared to other materials like HA, giving rise to a larger pool of available evidence from which more favourable outcomes are reported. Moreover, hyaluronic acid cannot be ruled out as a scaffold choice, and shows promising outcomes for success in REPs, but further clinical investigations are required to reliably evaluate its performance against other scaffolds.

### 6.1. Root Development Outcomes

Overall, a general trend identified across the reports was a superior outcome in root development with PRF compared to other scaffolds. However, it is also commonly suggested that regeneration can occur irrespective of scaffold type, indicating that other variables play a greater role in achieving success. Statistically significant outcomes were not consistent with root lengthening, although most cases experienced an increase. In contrast, improvements in dentin thickness had greater reproducibility. Prakash et al. (2023) proposed that a longer follow-up period was needed to show an increase in root length, implying that dentin thickening precedes root lengthening [25].

The root development success displayed with PRF could be attributed to its scaffold biology. Unlike PRP, which has a rapid release of growth factors, the dense fibrin structure of PRF provides a more stable scaffold with increased and sustained concentrations of growth factors, which play a vital role in promoting regenerative processes [25,27,29,33,36,37,39]. However, the lack of standardised preparation protocols across studies limits the comparability of these findings.

As well as the biological characteristics, the physical properties of scaffolds are also of major importance. Evidence suggests that the flexible fibrin framework of PRF is linked to enhanced improvements compared to PRP, as it forms a matrix that promotes cell movement within the canal space [36,39].

Greater root development in PRP compared to BCs was explained by similar reasons, with higher growth factor levels and consequently cellular activity. It has also been suggested that following disinfection, some stem cells, particularly stem cells of the apical papilla (SCAPs), may survive in the canal and differentiate into odontoblastic cells to form dentin [32,33]. Additionally, the stability, support, and release of growth factors from collagen scaffolds resulted in success, although PRF performance demonstrated greater effect due to a larger quantity of signalling molecules [27,35].

Promising results were also observed with HA, although greater development was observed with PRF. This may be attributed to its lower growth factor content, implying that it primarily acts as a biological framework, whereas PRF acts both as a scaffold and reservoir of proteins. Favourable results with HA have been attributed to its biocompatibility, hydrophilic properties, and resultant interaction with cell surface receptors [39]. This is further supported by other studies, which suggest that the slower degradation rate of HA facilitates dentin formation through prolonged structural support [23]. However, some authors proposed that dentin thickness appeared to increase due to deposition of cementum-like tissue [33], rather than true pulp tissue generation. This may suggest that whilst HA can provide good support, PRF may remain the clinical preference when the objective is to actively induce a regenerative response.

Heterogeneity in root development outcomes has been attributed to variability in patient characteristics, treatment protocols, and differences in follow-up periods, highlighting that successful results are not solely dependent on scaffold choice [24,34,37,38].

These findings were consistent with previous systematic reviews reporting that platelet concentrates, particularly PRF. may enhance regenerative outcomes due to their sustained growth factor release [14,41]. However, a recent review by Shahoon et al. (2025) reported that BCs produced significantly greater root length compared to PRP and PRF, which contradicts most existing results [42], emphasising the need for more trials evaluating comparative scaffold performance.

### 6.2. Apical Closure and Periapical Healing Outcomes

With respect to periapical healing and apical closure, findings were consistent with the trend observed in root development, presenting more consistent and effective outcomes with PRF compared to other scaffolds. Statistically significant findings were more frequent in apical closure outcomes compared to periapical healing reports, suggesting that soft tissue healing may be influenced by variables other than scaffold type.

Enhanced effects with PRF may be attributed to its bio-structural compatibility and prolonged biological activity. Its increased concentration of growth factors with sustained release promotes angiogenesis and osteogenesis, while the 3D fibrin network facilitates cell migration providing a stable regenerative microenvironment [29,30,33,37,39]. Additionally, its immunogenic properties, including higher concentrations of cytokines, have been associated with greater periapical healing [26,30,34,36].

Despite PRF outcomes being more consistent, PRP demonstrated superior periapical healing in the study by Shivashankar et al. (2017). This could be credited to its higher concentration of platelets and lower viscosity, which allows the material to easily reach the apex [40]. However, PRF appears more advantageous for apical closure due to its prolonged release of growth factors and capacity to increase collagen type I synthesis [26,37].

For both apical closure and periapical healing, hyaluronic acid’s favourable outcomes may be attributed to its ability to enhance cellular activity, particularly of SCAPs [39]. However, it is suggested that the material acts primarily as a bio-conductive scaffold, providing structural support in contrast to the bio-inductive properties observed in PRF and PRP, which may explain the distinction between scaffold types.

Moreover, healing outcomes may also be influenced by other factors, including variability in clinical protocols, as well as patient-related factors such as age, suggesting that periapical healing and apical closure may be less dependent on scaffold choice [25,37,38]. These findings are broadly consistent with previous regenerative endodontic literature reporting favourable healing and apical closure outcomes across different scaffold types [14].

### 6.3. Clinical Outcomes

The majority of included studies demonstrated high clinical success rates with most cases presenting patients as asymptomatic, regardless of scaffold group. However, it is important to distinguish between clinical survival and biological regeneration.

It has been suggested that this outcome is more dependent on other variables rather than scaffold selection, with multiple authors attributing success to standardised disinfection protocols [24,34,38], as well as patient-related factors such as apical foramen width, age, and tooth selection [34,38]. This implies that clinical success outcomes are mostly achieved through treatment technique and case selection rather than scaffold choice.

On the other hand, outcomes related to vitality had greater inconsistency and frequently presented negative results, indicating that clinical success does not necessarily result in regained pulp revitalisation. Variability in responses has been attributed to factors such as the position of MTA in the canal, which can produce an insulating effect [26,34,40]. Furthermore, authors agreed that vitality response is time-dependent, and it is suggested that follow-up periods of 12 months are insufficient for angiogenesis and neurogenesis to occur [26].

Negative vitality outcomes have also been associated with the formation of cementum-like tissue rather than true pulp tissue, giving rise to failed responses despite overall clinical success [35,37,40]. This suggests that current scaffolds may only repair rather than regenerate all odontogenic structures, and therefore perceived success should be reviewed with caution. These findings are similar to those of Yang et al. (2024), who described the current clinical evidence as inadequate due to many authors overlooking false negative responses [14].

### 6.4. Consistency and Statistical Significance

In the studies investigating PRF as a scaffold, more favourable outcomes were frequently observed compared to other materials, whilst those evaluating collagen and PRP identified improved results when compared to blood clots. Furthermore, studies evaluating HA showed promising results in relation to collagen and BCs, but literature remains limited. However, inconsistency exists between studies, and the available evidence reported cannot be used to make definitive conclusions on the most effective scaffold, despite the clinical trends observed.

Several reports observed statistically significant results between groups; however, significance was not reported consistently and varied depending on the outcome assessed. Many trials were also restricted to qualitative findings, limiting the overall strength and comparability of the evidence available.

In terms of root development, significant outcomes were more commonly reported for dentin thickening, which could be attributed to its earlier formation or greater radiographic detectability, whereas root length outcomes appeared to be time-dependent. Periapical healing was consistently observed, but reports of statistical significance were low. Conversely, apical closure demonstrated greater statistical significance, suggesting that this parameter may serve as a more reliable indicator of regenerative potential. Vitality was reported in few studies with most cases demonstrating negative responses, suggesting that it may represent a less reliable marker of success.

Generally, this review has identified that patterns between studies exist. However, the power of evidence is limited due to inconsistent statistical significance and should therefore be considered carefully. From a clinical perspective, scaffold choice alone may not determine treatment success, as disinfection protocols and patient-related factors appear to have a more critical role.

### 6.5. Limitations

Considerable limitations were found across the included studies in this review, which may have contributed to the outcome heterogeneity.

Issues in study design, including small sample sizes, restricted the strength of evidence. Follow-up duration is also important to consider, as outcomes such as vitality and root length are said to be time-dependent, limiting the reliable comparison of outcomes. Furthermore, variability in patient-related factors, such as pre-existing pathology, age, and apical foramen status, may have influenced results.

A lack of standardised clinical procedures was also evident. Although most trials used sodium hypochlorite and EDTA as irrigants, the concentrations and protocols differed, as did the intracanal medicament duration. Variability in treatment technique and material placement was also suggested to influence outcomes, particularly vitality and apical closure. Moreover, differences in PRP preparation may have affected its biological properties and contributed to inconsistent performance [37].

Differences in outcome assessment methods were also accountable for heterogenous results, with studies presenting findings as mean and standard deviations, categorical scoring systems, descriptive outcomes, and percentage changes, making comparisons between studies difficult. Furthermore, most studies were identified as moderate risk of bias or some concerns, primarily in domains related to randomisation, selective reporting, and outcome measurement.

Several limitations were present within this systematic review. Although a meta-analysis was initially intended, heterogeneity between studies made this unfeasible, and so a narrative synthesis was conducted limiting the ability to compare evidence between scaffold materials and compromising the quality of findings.

In addition, the limited number of studies investigating hyaluronic acid restricted comparisons, whilst PRF and PRP were more frequently evaluated. Moreover, findings by Turky et al. (2017), should be interpreted with caution, due to the journal’s unclear indexing status, which could present publication bias [32].

Overall, the methodological variability and limited available evidence restrict reliable comparisons. Therefore, conclusions of this review should be interpreted cautiously.

### 6.6. Future Research

In summary, it is evident that further trials with standardised methodology and assessment methods are required to make direct comparisons between scaffold groups. To draw definitive conclusions on the superiority and effectiveness of scaffold materials, higher quality reviews with longer follow-up periods are necessary.

Future research should aim to address the limitations of the current evidence, with particular focus on additional investigations using hyaluronic acid to allow comparisons with other scaffold materials, including PRP, PRF, and collagen.

Larger scale, well designed studies with standardised clinical procedures, such as irrigation and disinfection protocols, are needed to improve reliability of findings. Furthermore, future research should incorporate correct blinding, randomisation procedures, and complete reporting to minimise risk of bias.

## 7. Conclusions

To conclude, within the limitations of the available evidence, this systematic review does not indicate superior effectiveness of hyaluronic acid over PRP, PRF, or collagen scaffolds in regenerative endodontic procedures in immature necrotic permanent teeth. Platelet-rich fibrin (PRF) demonstrates the most consistent outcomes in terms of root development; however, apical closure and periapical healing appear to be less dependent on scaffold selection.

Hyaluronic acid shows promising biological properties, particularly in relation to cell proliferation and angiogenesis, but current clinical evidence remains insufficient to support its superiority over established scaffolds such as PRF or PRP.

Considerable heterogeneity in study design, outcome assessment and scaffold preparation protocols limited direct comparison between studies and reduced the strength of the conclusions.

Therefore, well designed, standardised randomised controlled trials with consistent outcome measures are essential to determine the true comparative effectiveness of scaffold materials in regenerative endodontics.

## Figures and Tables

**Table 1:** Summary of the keywords and Boolean operators used in each database.

Database	Keywords
PubMed	(“Regenerative Endodontics” OR revascularization OR revitalisation) AND (“Hyaluronic Acid” OR hyaluronan OR hyaluronate OR “Platelet-Rich Plasma” OR “Platelet-Rich Fibrin” OR “collagen scaffold” AND (“Pulp necrosis OR “necrotic pulp” OR immature OR “open apex”))
Scopus	(Regenerative endodontics) AND (Hyaluronic acid OR PRF OR PRP OR collagen) AND (Immature necrotic teeth)
EBSCO	(Regenerative endodontics OR Immature necrotic teeth OR Revascularization or Revitalisation) AND (Hyaluronic acid OR PRF OR PRP OR Collagen)
Google Scholar	“Regenerative endodontics” AND (Hyaluronic acid OR PRF OR PRP OR collagen) AND (immature teeth OR necrotic teeth)

**Table 2:** Summary of the study characteristics.

#	Author (s)	Year	Country	Study Design	Sample Size	Age Range	Tooth Type	Scaffold (s) Investigated	Follow-Up (Months)
1	Shivashankar et al. [40]	2017	India	Randomised controlled trial (triple-blind)	54 (PRF = 20, IB = 15, PRP = 19)	6–28 years	Immature necrotic permanent anterior teeth	PRP, PRF, induced bleeding	3, 6, 9, 12
2	Mazen et al. [23]	2021	Egypt	Randomised controlled clinical trial (2 arms)	30 (BCs = 15, BCsC and HA = 15)	Not reported	Immature necrotic permanent teeth	Group I: BCs (classic REP); Group II: BC and crosslinked HA gel	3, 6, 9, 12
3	El Sayed Sultan et al. [39]	2025	Egypt and Saudi Arabia	Randomised clinical trial	45 (PRF = 15, HA = 15, collagen = 15)	10–18 years	Immature necrotic permanent teeth	PRF, HA, collagen	3, 6, 12
4	Rizk et al. [24]	2020	Egypt	Randomised controlled trial (double-blind)	25 patients (PRP *n* = 13, PRF *n* = 12)	8–14 years	Immature necrotic permanent maxillary central incisors	PRP, PRF	3, 6, 9, 12
5	Ismaeal et al. [38]	2023	Egypt	Prospective clinical trial	30 teeth (BCs = 15, BCsC + Collagen = 15)	7–9 years	Immature necrotic permanent anterior teeth (trauma)	Group I:BCsCs; Group II: BC + collagen sponge	3, 6, 9, 12, 15, 18
6	Abo-Heikal et al. [37]	2023	Egypt	Randomised clinical trial	24 patients (i-PRF = 12, PRP = 12)	9–24 years	Immature necrotic maxillary central incisors (Trauma)	Group 1: Injectable PRF; Group 2: PRP	6, 12
7	Jiang et al. [35]	2017	China	Randomised controlled trial	43 teeth (40 patients) Control *n* = 22, experimental *n* = 21	Mean age: ~9.8–10.3 years	Immature necrotic permanent anterior teeth and premolars (pulp necrosis/apical periodontitis)	Group I: BCs (control); Group II: BC + Bio-Gide collagen membrane	6+ (control: Range of 8–28; Experimental range: 7–26)
8	Thakkar et al. [34]	2023	India	Retrospective comparative study	28 cases (BCs = 14, PRF = 14)	7–15 years	Immature necrotic permanent anterior teeth (trauma)	Group I: BCs; Group II: PRF	3, 6, 12
9	Narang et al. [36]	2015	India	Pilot randomised controlled trial	20 patients (5/group)	<20 years	Immature necrotic permanent teeth (caries/trauma)	Group I: MTA apexification (control); Group II: BCs; Group III: PRF; Group IV: PRP + collagen	6, 18
10	Prakash et al. [25]	2023	India	Randomised clinical trial	20 patients (10/group)	7–12 years	Immature necrotic permanent teeth (trauma/caries)	Group I: BC; Group II: PRF	1, 3, 6
11	Rizk et al. [33]	2019	Egypt	Split-mouth randomised controlled trial (double-blinded)	13 patients, 26 teeth (BCs = 13, PRP = 13)	8–14 years	Bilateral immature necrotic permanent maxillary central incisors	Group I: BC; Group II: PRP	3, 6, 9, 12
12	Turky et al. [32]	2017	Egypt	Randomised clinical trial	20 patients (BCs = 10, PRP = 10)	9–20 years	Immature non-vital maxillary anterior teeth	Group I: BC; Group II: PRP	6, 12
13	Khatab et al. [22]	2024	Egypt	Randomised clinical trial	14 patients (7/group)	9–12 years	Immature necrotic permanent anterior teeth	Group I: PRF; Group II: BCs + MTA	3, 6, 9,12
14	Prabhakar et al. [30]	2016	India	Non-randomised controlled clinical trial	14 patients (7/group)	10–12 years	Immature necrotic permanent teeth	Group I: BC; Group II: PRF	3, 6
15	Lv et al. [31]	2018	China	Retrospective controlled cohort study	10 patients (5 PRF, 5 BCs)	9–14 years	Immature incisors & premolars with apical periodontitis	Group I: BC; Group II: PRF	3, 6, 9, 12
16	Alagl et al. [28]	2017	Saudi Arabia	Randomised clinical trial (split-mouth design)	30 teeth (PRP = 15, BCs = 15)	8–11 years	Non-vital immature permanent teeth (24 maxillary incisors—trauma & 6 premolars—caries)	Group I: BC; Group II: PRP	3, 6, 9, 12
17	Rizk et al. [26]	2020	Egypt	Split-mouth randomised controlled trial (double-blinded)	24 teeth (PRF = 12, BCs = 12)	8–14 years	Bilateral immature necrotic maxillary central incisors	Group I: BC; Group II: PRF	3, 6, 9, 12
18	Mittal et al. [27]	2019	India	Randomised clinical study	16 teeth (4/group)	Not specified (paediatric cohort, immature teeth)	Immature necrotic permanent maxillary incisors with open apex	Group I: PRF; Group II: collagen; Group III: Placentrex; Group IV: Chitosan	3, 6, 12
19	Qamar et al. [29]	2025	India	Randomised clinical trial	28 teeth (A-PRF = 14; BCs = 14)	8–12 years	Non-vital immature maxillary incisors (trauma)	Group I: advanced PRF; Group II: BCs	3, 6, 12

**Table 3:** Comparative synthesis of radiographic and clinical outcomes according to scaffold material.

Scaffold	Root Length Increase	Dentinal Wall Thickening	Apical Closure	Periapical Healing	Evidence Strength
PRF	Most consistent improvement; frequently superior to PRP and BCs.	Most consistent improvement; commonly greater than PRP and BCs.	Favourable and frequently superior to PRP and BCs.	Generally favourable; often positive, although not always statistically significant.	Low—strongest trend among scaffolds but limited by heterogeneity and risk of bias.
PRP	Moderate improvement; often superior to BCs but less consistent than PRF.	Moderate improvement; several studies showed better results than BCs.	Positive closure outcomes; generally better than blood clots in some studies.	Good healing outcomes; one study reported superior healing compared with PRF.	Low—more evidence than HA/collagen, but inconsistent significance.
Hyaluronic acid	Promising but limited; one study showed PRF > HA > collagen, while another found no significant difference vs. BCs.	Promising; one study showed greater thickening than BCs, another found PRF > HA > collagen.	Favourable; significant results reported in limited studies.	Positive healing reported, mainly quantitatively.	Very low—evidence extremely limited, preventing definitive conclusions.
Collagen	Some positive outcomes, but generally less effective than PRF and HA when directly compared.	Improvement reported but limited and generally lower than PRF and HA.	Positive closure outcomes; some evidence of greater closure than BCs.	Positive healing reported but evidence remains limited.	Very low—limited number of studies and weak comparative evidence.

## Data Availability

The data presented in this study are available on request from the corresponding authors.

## Supplementary Materials

The following supporting information can be downloaded at: https://www.mdpi.com/article/10.3390/jcm15114257/s1, Table S1: PRISMA 2020 Checklist; Table S2: Grade Scale.
